# Data relating to factors affecting teachers’ burnout: A sem analysis in an Asian context

**DOI:** 10.1016/j.dib.2020.105448

**Published:** 2020-03-19

**Authors:** Lantip Diat Prasojo, Akhmad Habibi, Mohd Faiz Mohd Yaakob, Robin Pratama, Mat Rahimi Yusof, Amirul Mukminin, Suyanto, Farida Hanum

**Affiliations:** a Universitas Negeri Yogyakarta, Indonesia, Yogyakarta, 55281; b Universitas Jambi, Indonesia, Jambi, 36122; c Universiti Utara Malaysia, Malaysia, Kedah, 06010

**Keywords:** Dataset, Asian teachers, TSC, TE, burnout

## Abstract

The dataset presents the relationship between Teacher Self-Concept (TSC) and Teacher Efficacy (TE) as the predictors predicting burnout. Three components of burnout involved are Emotional Exhaustion (EE), Depersonalization (DP), and Reduced Personal Accomplishment (RPA). Various statistical approaches such as Content Validity Index (CVI), Exploratory Factor Analysis (EFA), Confirmatory Factor Analysis (CFA), Covariance-Based Structural Equation Modeling (CB-SEM) were addressed. Eight hundred seventy six Indonesian teachers form 3 provinces were willing to get involved by filling in the instrument. The data can be used for the educational institutions and centers to issue policies overcoming burnout among teachers, teachers to understand factors affecting their burnout, and future researchers extend the model offered by this dataset. This dataset is co-submitted from Heliyon entitled “Teachers’ burnout: A SEM analysis in an Asian context” [1].

Specifications table

**Table:** 

Subject	Social Science
Specific subject area	Educational psychology
Type of data	Tables Figures
How data were acquired	Content Validity Index (CVI), EFA, CFA, CB-SEM, Instruments: SPPS and AMOS
Data format	Raw Analyzed Filtered
Parameters for data collection	The data was distributed to Indonesian teachers. The instrument consists of Teacher Self-Concept (TSC), Teacher Efficacy (TE) and burnout; Emotional Exhaustion (EE), Depersonalization (DP), and Reduced Personal Accomplishment (RPA).
Description of data collection	The questionnaire was distributed to 1000 Indonesian teachers from 3 Indonesian cities; Jambi, Bandung, and Yogyakarta. Eight hundred and seventy-six responses were returned and analysed. Six hundered and eighteen (70.54%) are female respondents and 258 (29.35%) are males. Experience vary among teachers, 291 (33.22%) with less than five years experience and 585 (66.78) with more than five year experience.
Data source location	Jambi, Bandung, and Yogyakarta Indonesia 0.7893° S, 113.9213° E
Data accessibility	Repository name: Mendeley Data Data identification number: DOI: 10.17632/6jmv43nffk.2 Direct URL to data: https://data.mendeley.com/datasets/6jmv43nffk/2
Related research article	Prasojo, Habibi, Yaakob, Pratama, Yusof, Mukminin, Suyanto, Hanum (2020). Teachers’ burnout: A SEM analysis in an Asian context” HELIYON [1]

## Value of the data

• The dataset can be used for the educational institutions and centers to issue policies overcoming burnout among teachers
• Teachers, who play a significant role in educational development and experience burnout, can benefit the data to understand factors affecting their burnout.
• Future studies on teachers’ burnout can be conducted using this data

## 1 Data description

This dataset consists of 5 items of TSC from the TSC Evaluation Scale by [2]. Other 5 TE items were adapted from [3]. MBI items [4] were used for teacher burnout that consists of EE (5 items), DE (3 items), and RPA (7 items). A 5-point Likert scale ranging from 1 = never to 5 = always was addressed in the questionnaire. The adaptation was discussed with five educational experts for social and cultural adjustment. The decision of dropping some original items and changing the scales from a 7-point to a 5-point Likert was based on the discussion with the experts [5]. Various statistical approaches such as Content Validity Index (CVI), Exploratory Factor Analysis (EFA), Confirmatory Factor Analysis (CFA), Covariance-Based Structural Equation Modeling (CB-SEM) were addressed.

## 2 Experimental design, materials, and methods

The CVI involving 10 experts in educational psychology aimed at establishing the questionnaire validity. A 4-point scale in the CVI process (1 = not relevant/not clear to 4 = very relevant/very clear) were implemented [6] to measure the item level (I-CVI). The I-CVI reports a score of 3 or 4 by the experts that were divided by the total number of experts [7]. The I-CVI should not be lower than the value of 0.780 for 10 experts. As a result, one item was dropped due to lower value of the I-CVI. A pilot study with 50 Indonesian teachers was conducted after the CVI process. A reliability test was conducted by assessing Cronbach's alpha values; no values were reported to be lower than 0.700 as the threshold value [7].

The questionnaire was distributed to 1000 Indonesian teachers from 3 Indonesian cities; Jambi, Bandung, and Yogyakarta. Eight hundred and seventy-six responses were returned and analysed. Six hundred and eight teen (70.54%) are female respondents and 258 (29.35%) are males. Expereince vary among teachers, 291 (33.22%) with less than five years experience and 585 (66.78) with more than five year experience. The questionnaire version in English and Indonesian language can be accessed at http://dx.doi.org/10.17632/6jmv43nffk.2.

For the data preparation, Skewness and Kurtosis values of each construct were found to be normal ranging from −1 to +1 for the Skewness and −2 to +2 for the Kurtosis [7]. Using histogram, the data were reported to be normally distributed.

The process of the data was conducted through Cronbach's alpha to report the reliability. EFA and CFA were done for the factor analysis assessment. CB-SEM was addressed to assess the relationships between constructs.

For the EFA procedure, component principal analysis (PCA) approach was used to formulate uncorrelated linear combination against observable variables; Kaiser Meyer Olkin (> 0.500), Bartlett's Test of Sphericity (*p* < .05), eigenvalue (factor = > 1.0), communality (> 0.30), and factor loading (>. 40) [7,8]. The KMO value of the data was highly satisfactory (0.925) and Bartlett's Test of Sphericity was significant at *p* < .001. Using Varimax rotation, five factors were achieved with eigenvalues from 1.014 to 8.975. No issue emerged for the Communality. One item (EE5) was eliminated due to a highly cross-loading value. The Cronbach's alpha (α) values ranged from 0.754 to 875.

**Fig. 1:**
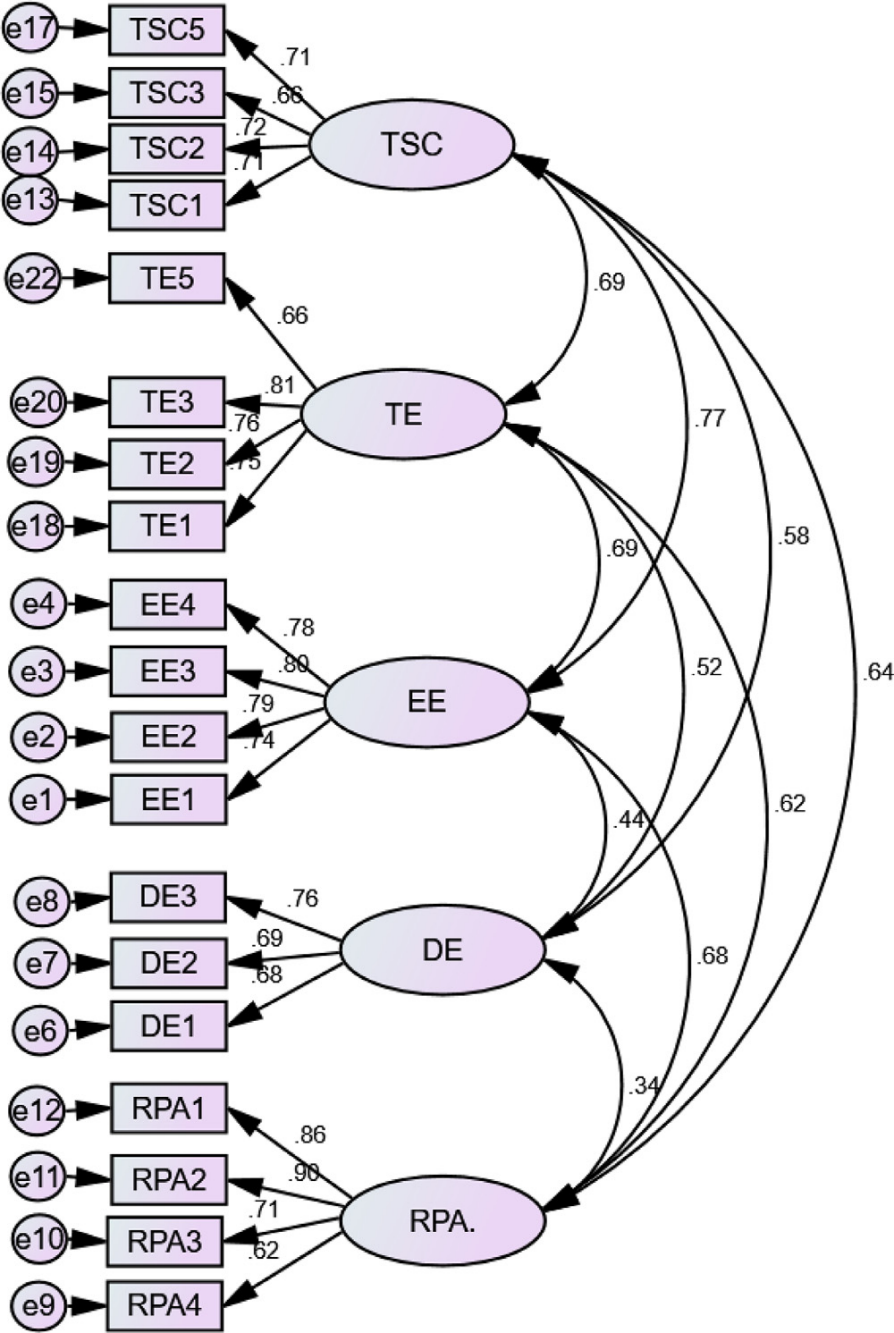
Confirmatory factor analysis.

Following the EFA procedure, CFA was examined using some indices [7] with their threshold values to confirm the EFA. The threshold values were implemented for each measurement; the Root Mean Square Error of Approximation (RMSEA = ≤ 0.08), Standardized Root Mean Square of Residual (SRMR = ≤ 0.08), Tucker-Lewis Index (TLI = ≥ 0.90), and Comparative Fit Index (CFI = ≥ 0.90). The initial measurement model did not meet the fit model due to TLI's low value. Two items, RPA5 & TE4, obtained lower loading values than the threshold; the items were eliminated. After the process, the measurement model was recomputed and meets all the threshold values (RMSEA = 0.07; SRMR = 0.02; TLI = 0.91; CFI = 0.93). The loading values ranged from 0.70 to 0.78 and Cronbach's alpha (α) values were from 0.75 to 0.86. Besides, average variance extracted (AVE) were also satisfactory from 0.75 to 0.86. Table 1 and Fig. 1 present the indices and values of CFA.

**Table 1:** Indices and values: CFA.

Indices	Values
χ2	830.37
χ2/df	5.19
RMSEA	0.07
SRMR	0.03
TLI	0.91
CFI	0.93

Similarly with the assessment of the measurement model, the structural model analysis through CB-SEM obtains satisfactory values (RMSEA = 0.071; SRMR = 0.02; TLI = 0.91; CFI = 0.92). Twenty indicators were included after the assessment of measurement and structural model for the final model.
